# Acute Obstructive Uropathy Complicated by Spontaneous Fornix Rupture and Hemorrhagic Urinoma

**DOI:** 10.7759/cureus.106683

**Published:** 2026-04-08

**Authors:** Mohammad Zaid, Omar A Al Mohdi, Rafe Alhayek, Abdulmunem M Alsadi, Fadhel A Yusuf, Yaser Saeedi

**Affiliations:** 1 Urology, Dubai Health, Dubai, ARE; 2 Medicine, University of Sharjah, Sharjah, ARE; 3 Urology, Dubai Hospital, Dubai, ARE

**Keywords:** obstructive hydronephrosis, obstructive uropathy, proximal ureteric stone, renal calyceal rupture, renal hemorrhage, ureteral stent

## Abstract

Acute ureteric obstruction is a common urological presentation, but complications such as spontaneous calyceal rupture leading to a urinoma and hemorrhage are rare. This report details the case of a 43-year-old male who presented with acute right flank pain and chills due to severe right hydroureteronephrosis caused by two large obstructive ureteric stones. Initial computed tomography (CT) of the kidneys, ureters, and bladder (KUB) revealed a large peri-renal fluid collection (approximately 6.9 cm) with heterogeneous hyperdense components (measuring approximately 47 Hounsfield units), consistent with a hemorrhagic urinoma secondary to renal fornix rupture. The patient’s clinical course was complicated by a significant drop in hemoglobin requiring transfusion. Immediate intervention via retrograde ureteral stent (6/26 double-J (DJ) stent) insertion successfully decompressed the system, resulting in clinical stabilization and a rapid reduction in urinoma size on follow-up imaging. Although the acute crisis was resolved, persistent cortical thinning of the right kidney and residual calculi necessitate a multi-stage management plan, including a dimercaptosuccinic acid (DMSA) scan to assess differential renal function and subsequent ureteroscopy laser lithotripsy for definitive stone removal. This case highlights the complexity of managing complicated obstructive uropathy and emphasizes the critical importance of prompt decompression to prevent irreversible renal damage.

## Introduction

Obstructive uropathy results from a diverse range of etiologies, categorized primarily into intrinsic factors, such as urolithiasis or transitional cell carcinoma, and extrinsic factors, including pregnancy, pelvic malignancies, or retroperitoneal fibrosis. These conditions create a mechanical barrier to urinary transit, resulting in stagnant flow and proximal collecting system distension. Pathologically, this condition occurs when intraluminal pressure rises significantly; if this pressure exceeds the capacity of the renal pelvis, it may lead to rare complications such as spontaneous fornix rupture [1,2]. This rupture results in urinary extravasation and the formation of a urinoma, a collection of encapsulated urine [3]. While iatrogenic rupture can occur during instrumentation, spontaneous rupture is a direct consequence of prolonged obstructive pressure and is considered a complex urological crisis [4]. Treatment primarily focuses on prompt decompression of the collecting system to preserve renal parenchyma, utilizing methods such as retrograde ureteral stenting or percutaneous nephrostomy. Barriers to effective management include delayed diagnosis and the presence of concurrent complications like hemorrhage or acute kidney injury (AKI), which are associated with long-term adverse outcomes [5,6]. Consequently, understanding the clinical context of hemorrhagic urinomas is vital for preventing irreversible renal damage. This case report presents a 43-year-old male with acute right ureteric obstruction complicated by a large hemorrhagic urinoma, emphasizing the critical importance of early intervention and a multi-stage management plan.

## Case presentation

A 43-year-old male with no significant past medical or surgical history presented to the emergency department with a one-day history of acute right flank pain and chills.

On admission, laboratory evaluations revealed a white blood cell count of 10.9 and an elevated C-reactive protein (CRP) of 2.9 mg/dL, indicating a systemic inflammatory state without meeting the threshold for leukocytosis. Significant renal impairment was present, characterized by a serum creatinine of 1.9 mg/dL and a corresponding estimated glomerular filtration rate (eGFR) of 43.5 mL/min/1.73 m², consistent with stage 2 AKI. Additionally, while the initial hemoglobin was 13.0 g/dL, a significant decrease was observed overnight, necessitating a blood transfusion.

The detailed laboratory results on admission are presented (Table 1).

**Table 1:** Initial laboratory findings on admission.

Test	Result	Standard Reference Range
White blood cell count	10.9	4.5-11.0 × 10⁹/L
Creatinine	1.93 mg/dL	0.7-1.3 mg/dL
C-reactive protein	2.9 mg/dL	<1.0 mg/dL
Hemoglobin	13.0 g/dL (on admission) then 10.0 g/dL after three hours from admission	13.5-17.5 g/dL (male)
Urinalysis	Microscopic hematuria	Negative for blood

**Figure 1:**
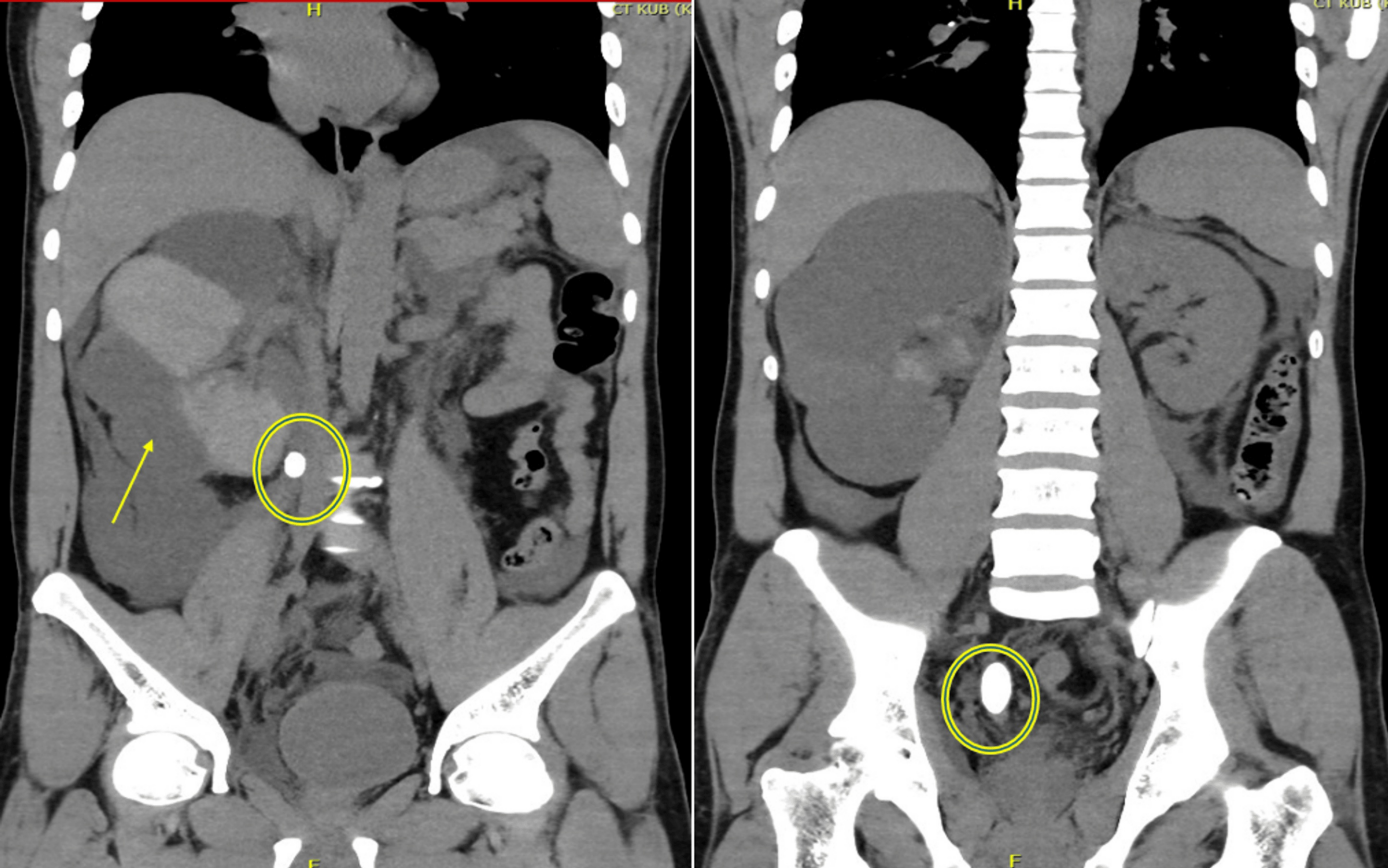
Coronal section of CT of the kidneys, ureters, and bladder (KUB). The arrowhead indicates severe right-sided hydroureteronephrosis and urinoma formation. The open circle highlights calculus in the upper ureter and the largest one, measuring about 1.8 cm.

Initial computed tomography (CT) of the kidneys, ureters, and bladder (KUB) demonstrated a large perirenal fluid collection (6.9 × 6.8 × 6.6 cm) with an average attenuation of 52 Hounsfield units (HU). These hyperdense components, compared to the expected water density of simple urine (0-20 HU), are diagnostic of a hemorrhagic urinoma. The presence of fluid-fluid levels was seen, further corroborating the presence of blood products secondary to the calyceal rupture (Figure 1).

**Figure 2:**
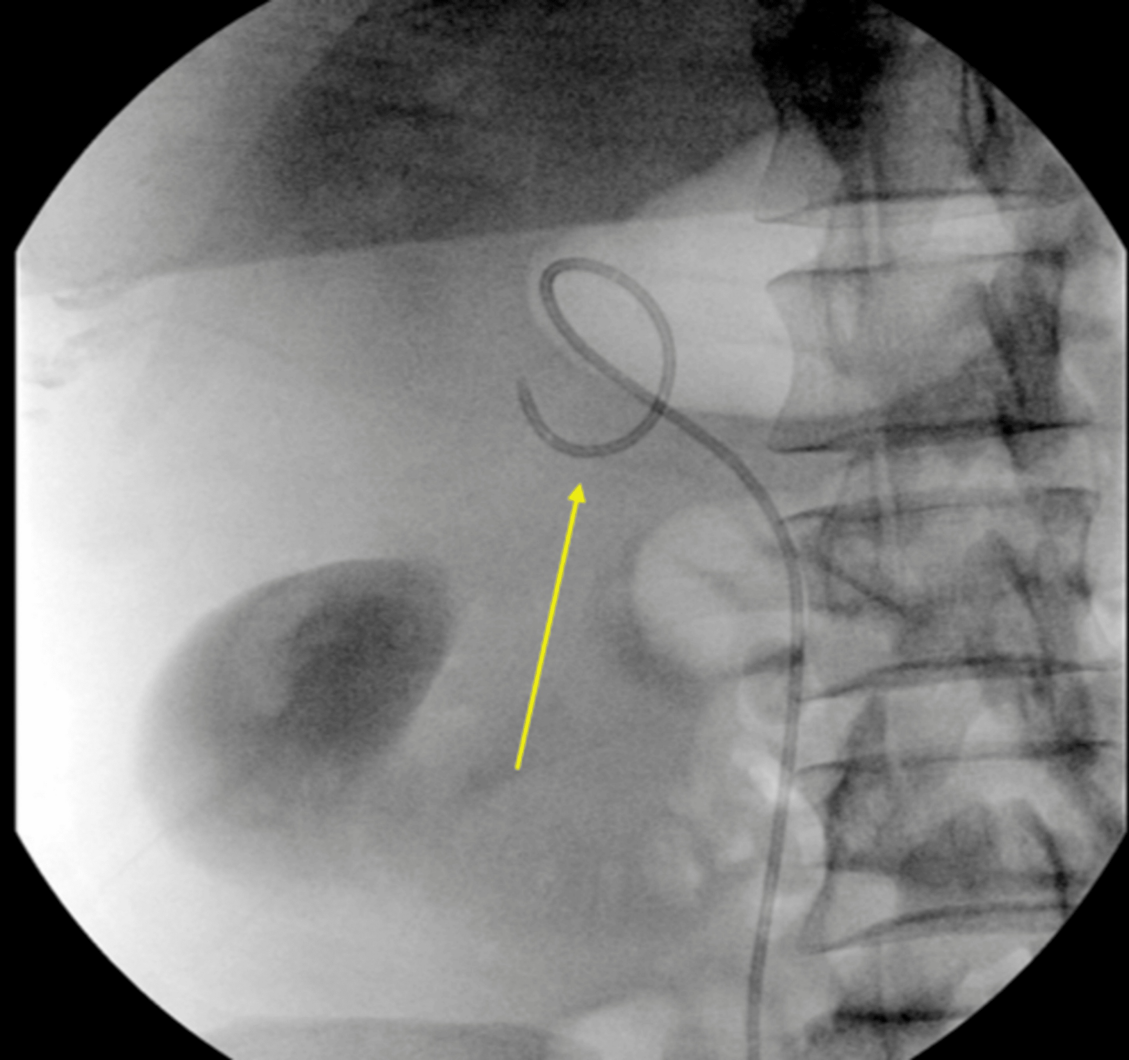
Fluoroscopy image shows the successful insertion of a 6/26 double-J stent (yellow arrow) into the right ureter. The double-J stent is visible, coiled in the renal pelvis and extending down the ureter.

On the same day, the patient underwent right ureteral stent insertion. Following cystoscopy access, a retrograde ureter pyelogram was initially performed to map the collecting system. This was followed by diagnostic ureteroscopy, which was indicated to directly evaluate the stability of the ureteric mucosa at the sites of stone impaction and to verify the integrity of the collecting system. Ureteroscopy confirmed the presence of two large, tightly impacted ureteric stones and a dilated upper tract. Direct visualization confirmed active urinary extravasation from the upper calyx, consistent with a fornix rupture. A 6/26 Fr double-J (DJ) ureteral stent was then successfully advanced under fluoroscopic guidance, bypassing both calculi to ensure adequate decompression and promote healing of the rupture (Figure 2).

**Figure 3:**
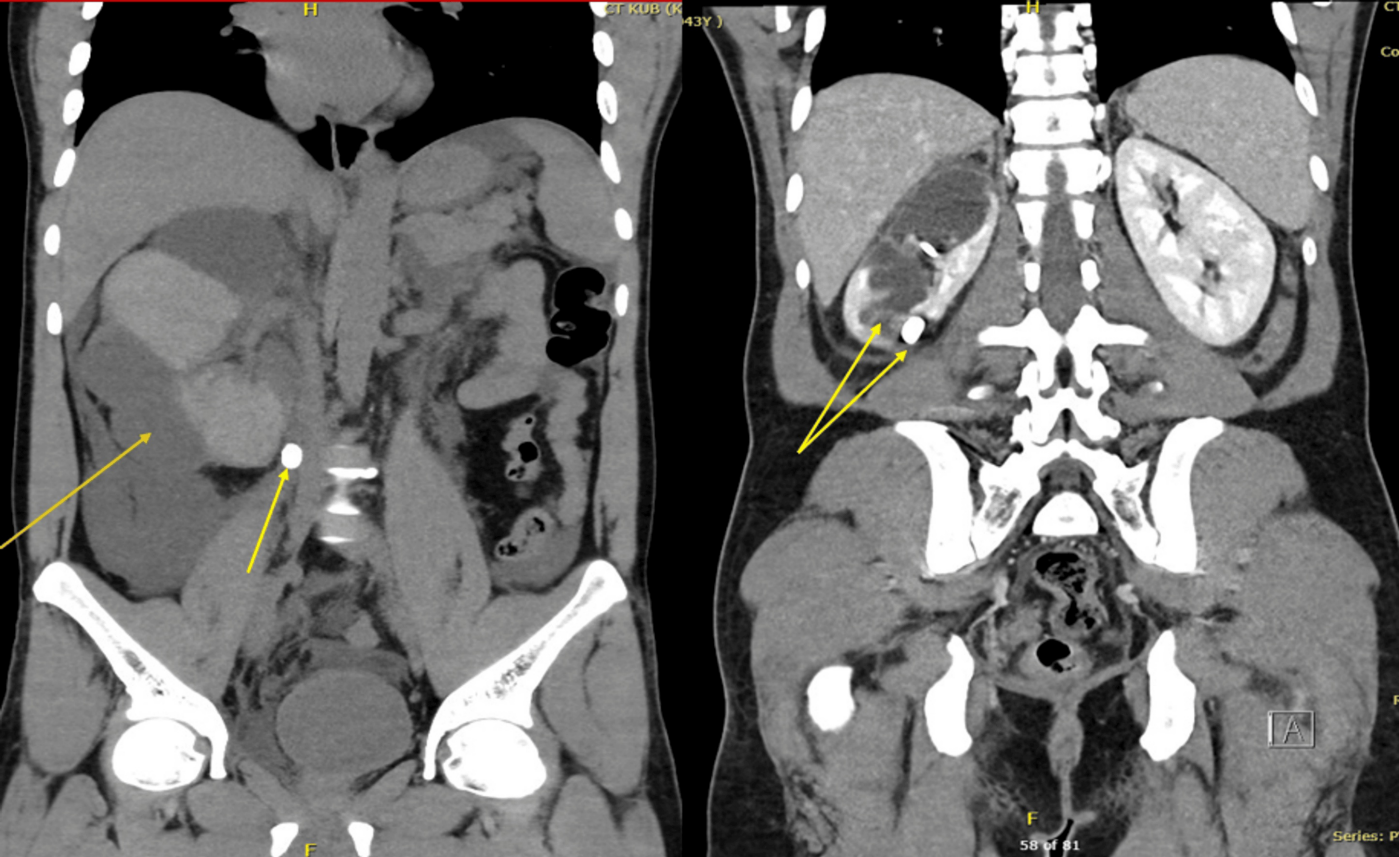
Two coronal-view CT scans show the abdomen before and after a medical procedure. The first scan reveals a large fluid collection (urinoma/hematoma), indicated by the left yellow arrow, and a proximal kidney stone on the right side (central yellow arrow). The second scan, a follow-up, demonstrates that the fluid collection has significantly reduced and the stone has moved into the lower part of the kidney (lower yellow arrow).

**Figure 4:**
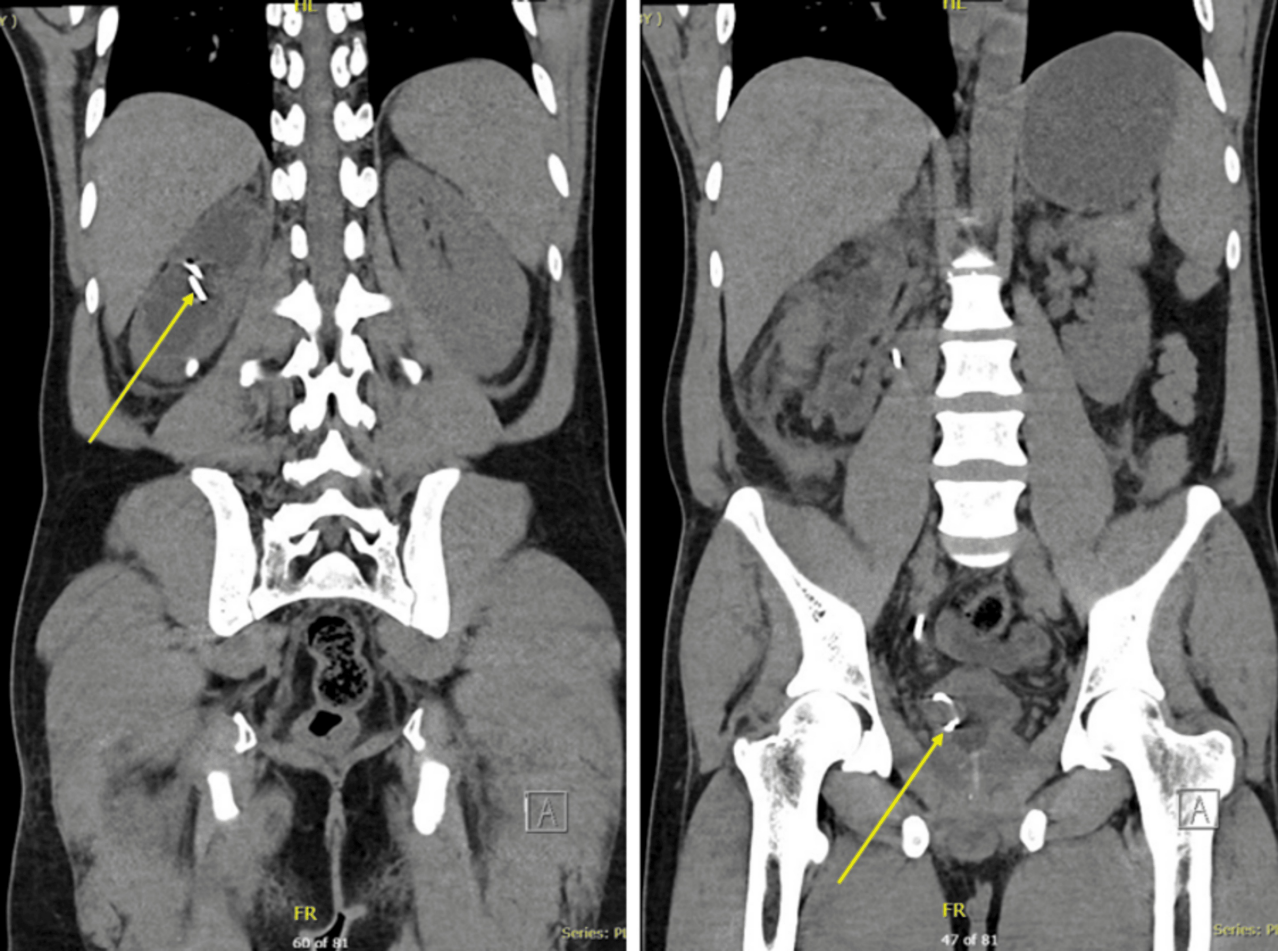
CT image demonstrating the in-situ ureteral stent (yellow arrow).

Given the systemic inflammatory features (chills and elevated CRP) and the high risk of infected obstructive uropathy, the patient was started on empiric intravenous piperacillin-tazobactam. The dosage was adjusted to 2.25 g every six hours to account for the patient’s AKI (eGFR 43.5 mL/min/1.73m². The antibiotic is the empiric choice for the hospital guidelines, and the 10-day course was completed as clinical markers of inflammation normalized. A repeat CT abdomen with contrast done a few days later demonstrated a grossly hydronephrotic right kidney with residual enhancing renal parenchyma (Figure 3). The large calculus previously noted in the proximal ureter had dislodged to the lower calyx (Figure 3). The urinoma/hematoma observed on the initial imaging had significantly reduced in size, with only mild fluid noted along the posterior pararenal fascia. Perinephric fat stranding and pararenal fascial thickening were also present (Figure 3). The right DJ stent was confirmed to be in a satisfactory position, while the large right distal ureteric calculus remained (Figure 4).

## Discussion

Acute obstructive uropathy from ureteral stones is a common urological emergency, but its complication by spontaneous renal fornix rupture is a rare and clinically complex event [1-3,7]. This patient's presentation, marked by severe hydroureteronephrosis, a large hemorrhagic urinoma, and AKI, exemplifies this severe but recognized complication. The rapid drop in hemoglobin requiring transfusion highlights the significant hemorrhagic component, which adds another layer of complexity to management [4,5].

The cornerstone of managing such cases is prompt decompression of the obstructed collecting system to relieve high intrarenal pressure, halt further urinary extravasation, and preserve renal function. The immediate insertion of a DJ stent proved to be a critical and highly effective intervention. Mechanistically, the stent facilitates decompression by acting as an internal bypass across the site of obstruction, providing a low-resistance conduit for urine to flow from the renal pelvis into the bladder. By rapidly reducing the intraluminal pressure below the threshold required for extravasation, the stent allows the forniceal tear to seal and the renal parenchyma to recover. As supported by the systematic review and meta-analysis by Zul Khairul Azwadi et al. [7], retrograde ureteral stenting is an established and efficacious method for managing acute upper obstructive uropathy. This intervention led to the patient's rapid clinical stabilization and the near-complete resolution of the large urinoma. While iatrogenic rupture can occur during retrograde manipulation [8], the spontaneous nature of the fornix rupture in this case was a direct consequence of the severe obstructive pressure [1-3].

Despite the successful management of the acute crisis, the follow-up imaging revealed persistent cortical thinning of the right kidney. This finding is clinically significant, as prolonged high-grade obstruction can lead to irreversible parenchymal damage and long-term adverse renal outcomes [5,9,10]. The presence of residual calculi, including the large distal ureteric stone and the now intrarenal proximal stone, necessitates a definitive, multi-stage management plan to prevent recurrent obstruction and further functional decline. The proposed plan, which includes a dimercaptosuccinic acid (DMSA) scan to quantitatively assess the differential function of the affected kidney, is essential for guiding subsequent treatment. Following this functional assessment, flexible ureteroscopy with laser lithotripsy is planned for complete stone clearance. This comprehensive approach aligns with best practices for managing complex urolithiasis, emphasizing both immediate stabilization and long-term renal preservation.

## Conclusions

This case report details a complex presentation of acute ureteric obstruction complicated by spontaneous calyceal rupture and urinoma with significant hemorrhage. The patient's clinical course underscores the importance of prompt diagnosis and intervention for this rare but severe complication. Early decompression via retrograde ureteral stenting was successful in stabilizing the patient's condition, leading to the resolution of the urinoma and improvement of inflammatory markers. While the acute emergency was managed, the presence of persistent hydronephrosis and retained stones highlights the need for a multi-stage approach. The long-term management plan, including a DMSA scan to evaluate differential kidney function and subsequent ureteroscopy laser lithotripsy, is crucial for preventing further renal damage and ensuring a favorable long-term outcome.
